# Ramping down a clinical 3 T scanner: a journey into MRI and MRS at 0.75 T

**DOI:** 10.1007/s10334-023-01089-9

**Published:** 2023-05-12

**Authors:** Christian Guenthner, Sophie Marie Peereboom, Hannes Dillinger, Charles McGrath, Mohammed Masoud Albannay, Valery Vishnevskiy, Max Fuetterer, Roger Luechinger, Theo Jenneskens, Urs Sturzenegger, Johan Overweg, Peter Koken, Peter Börnert, Sebastian Kozerke

**Affiliations:** 1 Institute for Biomedical Engineering, University and ETH Zurich, Zurich, Switzerland; 2 Philips Healthcare, Best, The Netherlands; 3 Philips AG, Zurich, Switzerland; 4 Philips Research, Hamburg, Germany

**Keywords:** Magnetic resonance imaging, Cine MRI, Multiparametric MRI, Technology assessment, Field strength

## Abstract

**Object:**

Lower-field MR is reemerging as a viable, potentially cost-effective alternative to high-field MR, thanks to advances in hardware, sequence design, and reconstruction over the past decades. Evaluation of lower field strengths, however, is limited by the availability of lower-field systems on the market and their considerable procurement costs. In this work, we demonstrate a low-cost, temporary alternative to purchasing a dedicated lower-field MR system.

**Materials and Methods:**

By ramping down an existing clinical 3 T MRI system to 0.75 T, proton signals can be acquired using repurposed ^13^C transmit/receive hardware and the multi-nuclei spectrometer interface. We describe the ramp-down procedure and necessary software and hardware changes to the system.

**Results:**

Apart from presenting system characterization results, we show in vivo examples of cardiac cine imaging, abdominal two- and three-point Dixon-type water/fat separation, water/fat-separated MR Fingerprinting, and point-resolved spectroscopy. In addition, the ramp-down approach allows unique comparisons of, e.g., gradient fidelity of the same MR system operated at different field strengths using the same receive chain, gradient coils, and amplifiers.

**Discussion:**

Ramping down an existing MR system may be seen as a viable alternative for lower-field MR research in groups that already own multi-nuclei hardware and can also serve as a testing platform for custom-made multi-nuclei transmit/receive coils.

**Supplementary Information:**

The online version contains supplementary material available at 10.1007/s10334-023-01089-9.

## Introduction

In recent years, magnetic resonance imaging at lower fields (0.1 T ≤ B_0_ ≤ 1 T) has received renewed interest [1–7﻿]. This development is driven in parts by the necessity to reduce procurement and operation costs of MR systems in healthcare markets. As the magnet accounts for more than 30% of the overall system cost [8], lowering the static field has been pursued as a cost-cutting option in conjunction with the design of sealed cryo-systems requiring minimal liquid helium to reduce installation and operation expenses. Lowering the field strength is beneficial in relation to specific absorption rate (SAR) limits, as higher transmit-field strengths at lower static field allow for reduced radiofrequency pulse durations and hence longer sampling windows for a given repetition time as well as permitting higher flip angles in balanced and spoiled field-echo protocols. At the same time, longer readouts are also supported by the increase of T_2_* at lower field, which allow recouping some of the signal-to-noise ratio (SNR) losses by reducing the readout bandwidth [9]. Reduced T_1_ times in conjunction with larger flip angles in balanced and spoiled field-echo protocols further allow to reduce these losses [10]. Moreover, the impact of the magneto-hydrodynamic (MHD) effect [11] is reduced at lower field, which makes ECG-triggering or gating more reliable. Finally, from the perspective of patient comfort, acoustic noise is lowered due to reduced Lorentz forces between the cryostat and the gradient coils [12].

Since 0.5 T systems entered clinics in the 1980s, significant advances in magnet technology, gradient and radiofrequency hardware, pulse sequence design and image reconstruction have been achieved, now rendering lower-field systems potentially competitive with current high-field systems for several applications [13]. For example using a 0.35 T MRI split-bore scanner integrated into a radiation therapy setup, the value of lower fields for cardiac imaging application was pointed out early [14, 15]. Meanwhile, a number of 0.55 T systems have been deployed at several sites. The first of these installations, at the National Institutes of Health (NIH), was used to demonstrate excellent image quality for cardiac, body, lung, and interventional imaging [2]. This and earlier work sparked our interest in evaluating lower-field imaging [14–18] for cardiac [17–22] and quantitative body imaging applications [23, 24]. To keep time and cost overheads to a minimum, it was decided to temporarily ramp down a clinical 3 T system with high-performance gradients and utilize the existing ^13^C transmit/receive hardware to study proton MRI and MRS at 0.75 T. Contrary to similar ramp-downs by the NIH, University of Southern California, and the New York University, the 3 T body transmit/receive coil remained in the scanner, making our installation reversible and relatively quick to perform (1/2 day per ramp-down/-up) and by repurposing existing hardware, overall costs were reduced. In the present manuscript, we give a detailed account of the ramp-down procedure on both a hardware and software level, show system characterization results, and present several in vivo MRI and MRS use cases.

## Methods

The ramp-down was performed on a 3 T Philips Achieva scanner (Philips Healthcare, Best, the Netherlands) with a dual-amplifier gradient system delivering 200 T/m/s slew rate at a maximum gradient strength of 40 mT/m or 100 T/m/s at 80 mT/m.

### Choice of field strength

To use the 3 T multi-nuclei hardware for transmission and reception of the proton signal at the reduced field strength ${B}_{0}$, the resonance frequency of the existing ^13^C transmit and receive coils had to match the Larmor frequency of protons. Using the gyromagnetic ratio of ^13^C (${\gamma }_{13C}=67.28$ rad MHz/T) and of ^1^H (${\gamma }_{1H}\approx 267.52$ rad MHz/T), the Larmor frequency is given by

$${\gamma }_{13C}\cdot 3T={\gamma }_{1H}\cdot {B}_{0,}$$

leading to

$${B}_{0}=\frac{{\gamma }_{13C}}{{\gamma }_{1H}}\cdot 3T\approx 0.251\cdot 3T\approx 0.75T.$$

**Fig. 1:**
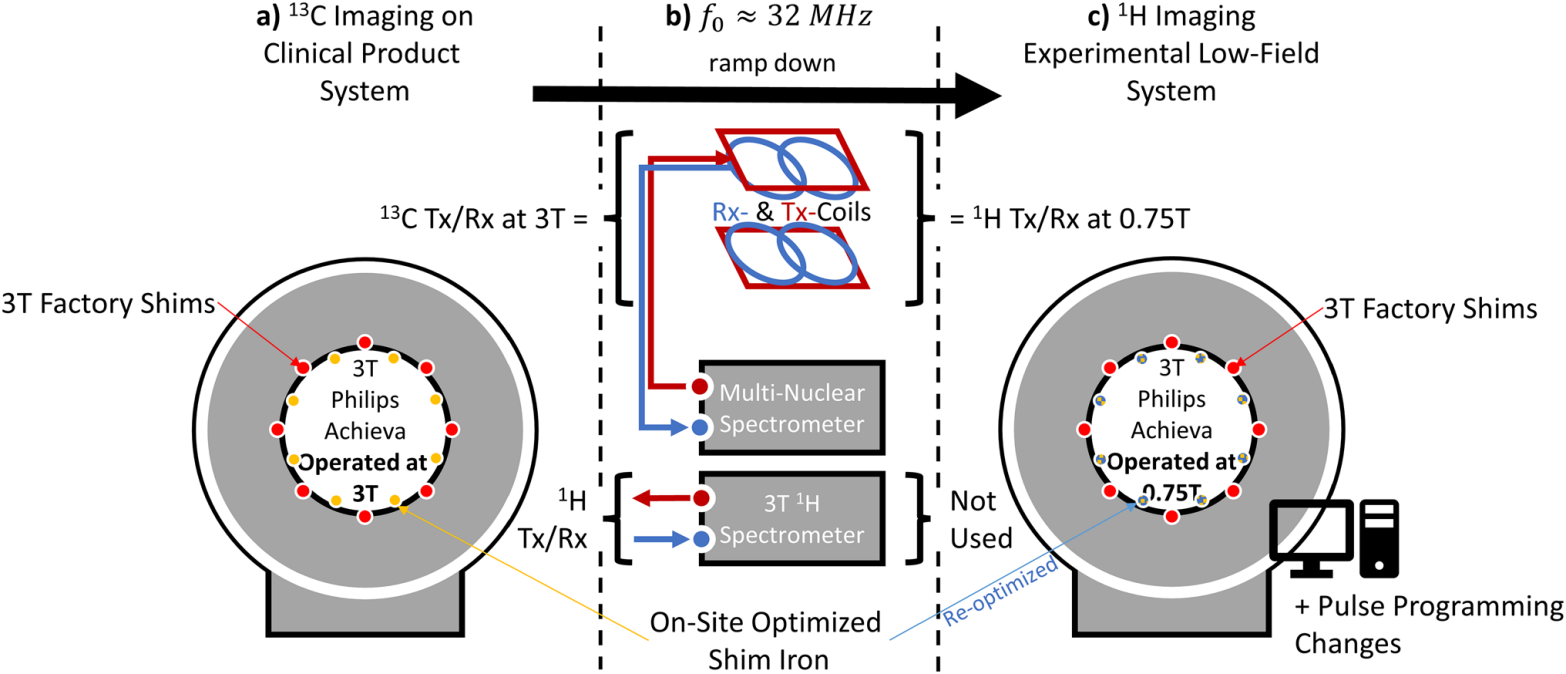
Schematic of the ramp-down experiment. To receive proton signals at a reduced field strength using existing ^13^C transmit/receive (*T*x/*R*x) hardware (**b**), a clinical 3 T system (**a**) was ramped down to 0.75 T (**c**). In both configurations, the Larmor frequency is equal as the gyromagnetic ratio of ^1^H is approximately four times larger than that of ^13^C. As the scanner software remained configured for 3 T, pulse-programming software changes had to be made to account for the difference in Larmor frequency, gyromagnetic ratio, and absence of a body coil tuned to 32 MHz. The 3 T body coil remained connected inside the scanner. Factory shims (red) are bolted to the cryostat and could not be removed. Shim rails with site-specific shims for 3 T operation (yellow) were removed and replaced by a re-optimized set for 0.75 T operation (blue)

Hence, the target field strength of 0.75 T was chosen. A schematic of the ramp-down procedure and required modifications is shown in Fig. 1.

### Pulse-programming changes

The system’s software had to be changed to enable proton imaging at the reduced field strength. From the software perspective, the scanner remained a 3 T transmitting and receiving at the ^13^C Larmor frequency. However, gradient fields were calculated using the proton gyromagnetic ratio instead to enable proton imaging at 0.75 T. The steps were as follows:

1. To calculate the correct gradient fields for imaging, the gyromagnetic ratio of ^13^C was overwritten with the value for protons ${\gamma }_{1H}$.
2. After Larmor frequency determination, the frequency is checked to lie within a narrow range of the expected values based on the 3 T field strength and the proton gyromagnetic ratio. These checks had to be disabled, otherwise the system would refuse to scan.
3. Preparation phases had to be adapted to use the local receive coils instead of the body coil. Pick-up coil-based preparation phases had to be disabled as these would have operated on the wrong resonance frequency.
4. Since parallel imaging (SENSE) and compressed sensing (CS-SENSE) scan features require a body coil reference scan for determining sensitivity maps, additional modifications were required given that the former 3 T body coil was not operational:
  - The SENSE reference scan had to be adapted to run without a body coil. Here, the second acquisition that utilizes only the body coil for reception was adjusted to use a single receive coil element. These reference scans were only required to allow running SENSE/CS-SENSE accelerated scans and were automatically used by the vendor’s reconstructor to produce images on the console. For offline reconstruction, the acquired SENSE reference data were disregarded and coil sensitivities were estimated separately.
  - SENSE/CS-SENSE feature checks had to be overwritten to run without a body coil and with a non-SENSE-certified coil.
5. For water/fat separation using multi-echo and multi-acquisition protocols (DIXON), water/fat shift calculation had to be adapted to work with the resonance frequency determined during the preparation phases instead of using predefined field strength-dependent settings.

### Hardware modifications

#### Ramp-down

The 3 T magnet was ramped down three times between 2019 and 2021. As the critical temperature of the super-conductor (SC) increases with decreasing field strength and self-heating is reduced due to the lower current density at lower magnetic field, the SC heater had to be tested to ensure that the magnet could be safely taken off field. Hence, a quench test was performed at 0.75 T during the initial ramp-down trial. Using the 3 T quench system, the field strength could be reduced to a value below 10 mT within 2 min as compared with 20 s for the product field strength. This was deemed safe given that only specially trained personnel were allowed to enter the MR room.

#### Re-optimization of shim irons

The 3 T system utilizes two sets of shim irons, (1), a factory shim set, which is bolted to the cryostat, and (2), a removable shim set, which can be optimized on-site using a static-field camera and a simulation tool provided by the vendor. For the 0.75 T configuration, the removable shim irons were re-optimized to compensate both the intrinsic inhomogeneity of the magnet at 0.75 T as well as the field generated by the fixed 3 T factory shims. A main field homogeneity of 1.3 ppm root-mean-squared (RMS), equal to 42 Hz RMS, was achieved within a 40 cm sphere. The homogeneity in the standard 50 × 50x45 cm^3^ volume was 4.7 ppm RMS (150 Hz RMS) and, thus, considerably lower than the relative specification for 3 T (≤ 3 ppm RMS), due to limited space on the shim rails to compensate for the bolted shim set. In absolute frequency terms, the lower-field configuration provided a roughly 2.5-fold better field homogeneity within the standard 50 × 50 × 45 cm^3^ volume compared to the minimum requirements of the system at 3 T (absolute: ≤ 383 Hz RMS). Only within a narrow 20 cm sphere could the system be shimmed within industry requirements (≤0.04 ppm) providing a true fourfold increase in field homogeneity.

#### RF coils

The 3 T proton body coil could not be used and remained connected inside the scanner in a de-tuned state. For transmission and reception, an existing ^13^C jacket double Helmholtz pair (Clinical MR Solutions, Brookfield, WI, USA) was employed as a transmitter coil, while an existing ^13^C four-channel cardiac receive array (Clinical MR Solutions, Brookfield, WI, USA) with two posterior and two anterior rectangular elements (7.6 cm x 18.3 cm) was used for signal reception. Both coils pre-existed and were used in ^13^C experiments at 3 T before. The transmit coil was tested and calibrated on a standard load phantom. The maximum transmit-field strength (B1 +) was limited to 45 mT. To ensure safe operation in the in vivo setting, electromagnetic (EM) fields produced by coils and cables were measured and checked using a dedicated EM exposure acquisition system (EASY4MRI, Schmid & Partner Engineering AG, Zurich, Switzerland).

### Costs of operation

The following provides an approximate list of costs to pursue the ramp-down experiment. Approximate costs are listed in Swiss Francs (CHF).

- **Static field probes (12.5 k CHF):** Magnetic field measurement device to map out the static field to optimize shim iron placement.
- **Shim rails (5 k CHF):** A set of shim rails were purchased to simplify switching between field strengths. With these, the 3 T shim rails could be removed after ramp-down of the magnet and replaced by the dedicated lower-field shim sets, before bringing the system to its final 0.75 T main field strength and vice versa.
- Ramping down and ramping up (12 work hours of Philips engineers per ramp-down cycle): After the initial ramp-down test, each successive ramp-down took approximately six hours for one service engineer and additional six hours for ramping up.
- **Helium (~ 50 l per ramp-down):** Including the quench test in the initial ramp-down, a total of 150 l of Helium was purchased over the course of the three ramp-downs. As Helium is captured during ramp-down, boil-off is minimal and 50 l/ramp-down should be seen as an upper bound.
- **Transmit and receive coils (0 CHF):** No direct costs arose from the purchase of T/R hardware as the existing 3 T ^13^C hardware could be repurposed.
- **Indirect Costs:** Work hours by our scientists to make scanning possible cannot be directly quantified and can hardly be disentangled from the research work that has been performed on the system.

**Total:** We estimate a total investment of approximately 100 k CHF over the whole project including own work hours.

### Acquisition, data processing and reconstruction

The ramped-down system was used as a test platform for a variety of scan techniques and hardware tests. Apart from mapping static, transmit, and receive fields, gradient modulation transfer functions and audio response functions were obtained. In vivo data were acquired on healthy subjects in accordance with institutional and ethical guidelines and upon informed consent. A detailed list of scan parameters can be found in Table 1.

**Table 1:** List of scan parameters

Scan type	Sequence	Readout (AQ duration, notes)	Field Strength	TR (ms)	TE (ms)	Flip angle	No aver	FOV (mm^2^)	Slice thickness	Matrix size	Voxel Size (mm^2^)	No. BH x Dur	Notes
B0 mapping	2D ME T1w-FFE	Spiral (5.0 ms, 40 Interleaves, 2 Echoes)	0.75 T	35.0	0.9 (ΔTE 9.2)	15°	6	350 × 80	8 mm	176 × 40	1.98 × 2.00	1 × 17 s	Iterative B0 Estimation and MFI Deblurring
B1 + mapping	DREAM	Cartesian (918 μs)	0.75 T	3.7	1.0 (SE) 2.2 (STE)	20°	10	350 × 350	8 mm	72 × 72	4.86 × 4.86	1 × 18.3 s	STEAM Preparation Angle: 50°
	DREAM (for MRF)			4.0	1.1 (SE) 2.6 (STE)								STEAM Preparation Angle: 90°
Cardiac CINE	2D bSSFP	Cartesian (2.6 ms)	0.75 T	4.3	2.1	60°	1	350 × 350	10 mm	176 × 176	1.99 × 2.06	1 × 18 s	22 Cardiac Phases/ECG Gating
		Cartesian (660 μs, SENSE: 2.2, Rect. FOV: 125%, Fold-Over Suppression:$\pm$ 20%)	1.50 T	2.64	1.3			299 × 372	8 mm	176 × 219	1.70 × 1.75	1 × 7.6 s	36 Cardiac Phases/ECG Gating
		Cartesian (500 μs, SENSE: 2.2, Rect. FOV: 125%, Fold-Over Suppression:$\pm$ 20%)	3.00 T	4.41	2.2			299 × 372	8 mm	176 × 219	1.70 × 1.75	1 × 14.2 s	36 Cardiac Phases/ECG Gating
Dixon water/fat separation	MA T1w-FFE	Cartesian (2.6 ms, 3 Echoes)	0.75 T	11.5	2.9 (ΔTE 3.1)	15°	4	350 × 350	8 mm	176 × 176	1.99 × 1.99	1 × 25.7 s	Iterative Water/Fat/B0 Estimation
	ME T1w-FFE	Cartesian (2.0 ms, 3 Echoes)		12.1	4.0 (ΔTE 3.1)							1 × 10.7 s	Iterative Water/Fat/B0 Estimation
	MA T1w-FFE	Spiral (10 ms, 22 interleaves, Archimedean, 3 Echoes)		18.8	1.1 (ΔTE 3.1)							1 × 6.5 s	Iterative Water/Fat/B0 Estimation and MFI/Fat-Spectrum Deblurring
	MS ME FFE	Cartesian (3.8 ms, 2 Echoes)	0.75 T	133.4	TE1: 4.6 TE2: 9.2	75°	1	325 × 250 × 219 mm	10 mm (Δ: 1 mm)	212 × 130 (20 Slices)	1.53 × 1.93	2 × 18 s	Iterative Water/Fat/B0 Estimation
Water/fat-separated MRF	Dual-TE FISP-MRF	Spiral (12 ms, 14 interleaves, effective spatial undersampling factor: 7, Archimedean)	0.75 T	25.0	TE1: 4.6 TE2: 9.2	0–60°	1	350 × 350	8 mm	176 × 176	1.99 × 1.99	1 × 25 s	Adiabatic Inversion, 1000 Shots 7 Peak Fat-Spectrum Deblurring in k-space, multi-frequency interpolation
Balanced SSFP	M-2D bSSFP	Cartesian (3.4 ms)	0.75 T	4.7	2.3	75°	1	325 × 275 × 164 mm	10 mm (Δ: 1 mm)	208 × 131 (15 Slices)	1.56 × 2.10	1 × 17 s	
Spectroscopy	PRESS	Spectral (512 ms, Bandwidth: 1 kHz, 512 Samples)	0.75 T	≥ 2000	15	90°	16 (192)	n/a	n/a	1 × 1×1	10 × 20×0 mm (8 ml)	n/a	16 × Water Unsuppressed Spectra 192 × Water Suppressed Spectra ECG-Triggered Respiratory Navigator Gating
		Spectral (256 ms, Bandwidth: 2 kHz, 512 Samples)	1.5 T		18								
		Spectral (128 ms, Bandwidth: 4 kHz, 512 Samples)	3 T		26								
GMTF	MS FFE	Spectral w/concurrent gradient chirp (128 ms, Bandwidth: 512 kHz)	0.75 T	560	2.7	90°	5	252 × 252 × 110 mm	2 mm Δ: 34 mm	7 × 7×4	36 × 36	13:43 min	Chirp: Concatenated Sinusoids, 100 Hz to 10 kHz Sweep, Positive/Negative Polarity along Slice-Encode Direction
			3 T		2.8			180 × 180 × 110 mm		5 × 5×4		7:16 min	

Reconstruction using the vendor’s reconstructor was possible for most of the acquired data producing images for planning and initial data evaluation on the console. As no coil sensitivity prescans were available, coil-combined reconstructions showed artifacts from phase differences between the receive elements. Hence, all raw data including scan metadata were exported using ReconFrame (GyroTools LLC, Winterthur, Switzerland) and reconstructed offline with MRecon (GyroTools LLC, Winterthur, Switzerland) in MATLAB 2020b (Mathworks, Natick, Massachusetts, USA) and the Berkley Advanced Reconstruction Toolbox (BART; Berkley, California, USA) [25].

Unless otherwise stated, coil sensitivities $S$ were determined using BART’s ecalib [26] implementation with the option ‘m1’ to produce only the first sensitivity map and ‘c0’ to disable cropping of the maps. To homogenize image contrast, the magnitude of the Roemer coil-combined images [27] was blurred by a 20 px Gaussian filter to capture primarily the low-frequency contrast variations produced by the receive coils. This provided a fake quadrature body coil (QBC) image $Q$, which was used to regularize the coil combination on a per-pixel basis. Per-pixel, the coil-combined image $I$ was calculated usin﻿g [28]

$$I = \left( {S^{\dag } S + \lambda Q^{p} } \right)^{ - 1} S^{\dag } i,$$

where ${\varvec{i}}$ is the vector of single-coil reconstructions, $\dag$ is the Hermitian conjugate. The power $p$ = 1 and the regularization parameter $\lambda$ = 0.3, where chosen manually to compromise between background signal suppression and contrast homogenization.

All spiral scans were deblurred using multi-frequency interpolation with 19 equidistant demodulation frequencies between − 300 and + 150 Hz (25 Hz steps) [29]. B0 maps for deblurring were either obtained from the data itself (B_0_ Mapping and Dixon Multi-Acquisition scans) or prescribed (MRF scan).

Reconstruction code and raw data for all imaging experiments are available for download on gitlab https://gitlab.ethz.ch/ibt-cmr-public/recon-0.75t-mri.

### System characterization

#### B_0_ mapping

Main field (B_0_) mapping was performed in a transversal and coronal, abdominal slice using a dual echo time, spoiled gradient-recalled echo (GRE) sequence with a spiral readout. Spiral deblurring and field-map estimation was performed in a self-consistent, iterative fashion. In each iteration, the estimated B0 map was first unwrapped in 2D and then blurred using an adaptive 2D Gaussian filter with decreasing filter strength from 60 to 10 px in 10 steps followed by 10, 8, 6, 4, 2, 1 px in subsequent iterations. Due to the strong signal attenuation in the transversal orientation, the fake QBC image was calculated using a reduced Gaussian filter of 10 px for better contrast homogenization.

#### DREAM B1 + mapping

For B1 + mapping, the DREAM sequence [30] was used in both transversal and coronal angulations.

#### Receive field mapping

Approximate receive field maps were obtained from the dual-TE B0 mapping scan by reconstructing each coil image separately and dividing it by their sum of squares (SOS). In addition, a homogenized SOS image was obtained by dividing the SOS by its Gaussian-blurred ($\sigma$ = 20 px) image.

#### Gradient modulation transfer function (GMTF)

The GMTF was measured using the chirp-based spectroscopy approach by Rahmer et al. [17, 31]. In short, in parallel to a thin-slice multi-slice spectroscopy acquisition with in-plane phase-encoding, a chirp pulse was played out in through-slice direction with both positive and negative polarity. The acquisition volume was rotated to obtain measurements of the three cardinal axes. After filtering of low-signal regions, the phase-time course of each voxel was numerically differentiated and fit by spherical harmonics. By dividing their spectrum by the input chirp spectrum, an estimate of the gradient modulation transfer function was obtained.

#### Relative microphone amplitude spectra (RMAS)

Concurrent to the GMTF measurement, audio measurements using a microphone (Sennheiser ME 66, Wedemark, Germany), placed into a waveguide of the MR Faraday cage, and a linear audio recorder (Tascam DR-100) were performed on both field strength configurations (0.75 T and 3 T). To calibrate microphone levels between ramp-down and product field strength, a reference sound source at 1 kHz was used. Equivalently to the GMTF, the audio spectrum was divided by the chirp spectrum to obtain the relative microphone amplitude spectra (RMAS), a measure of the frequency-dependent acoustic noise emissions allowing direct comparison of the system in its low-field and product field strength configuration.

### In vivo showcases

#### Cardiac cine imaging

Cardiac cine scanning was performed at three field strengths, i.e., at 0.75 T, 1.5 T, and 3 T. While measurements at 0.75 T and 3 T were conducted on the same scanner, a separate 3 T Philips Achieva system (Philips Healthcare, Best, the Netherlands), which is permanently ramped down to 1.5 T, was used for the experiments at 1.5 T. Fully sampled 2D balanced steady-state free-precession (bSSFP) cine images were obtained in short-axis, long-axis, and four-chamber views using retrospective vectorcardiogram (VCG) gating.

In addition, the acquired k-space data of the 0.75 T short-axis cine scans was retrospectively undersampled in k–t space using a variable density sampling mask and reconstructed using a cyclic vectorial total variation regularization [32] approach to demonstrate achievable image quality and acceleration factors with modern reconstruction techniques [19]. We minimized the following optimization problem

$$\underset{{\varvec{\rho}}}{\mathrm{min}}{\Vert \widehat{M}\widehat{F}\widehat{S}{\varvec{\rho}}-{\varvec{d}}\Vert }_{2}^{2}+\lambda {\Vert \left[\alpha {\widehat{\nabla }}_{\mathrm{x}}{\varvec{\rho}},\alpha {\widehat{\nabla }}_{\mathrm{y}}{\varvec{\rho}},{\widehat{\nabla }}_{\mathrm{t}}{\varvec{\rho}}\right]\Vert }_{\mathrm{2,1}},$$

where ${\varvec{\rho}}$ represents the frames of the reconstructed cardiac cine, $\widehat{S}$ is a coil sensitivity operator, $\widehat{F}$ the Fourier transformation, $\widehat{M}$ the undersampling mask, and ${\varvec{d}}$ the acquired data. For regularization, $\alpha = 0.05$ was used to balance spatial variation ${\widehat{\nabla }}_{x,y}{\varvec{\rho}}$ against temporal variations ${\widehat{\nabla }}_{t}{\varvec{\rho}}$. Optimization was performed numerically using the alternating directions method of multipliers (ADMM) [33]. Inner quadratic optimization was performed using three conjugate gradient steps. The regularization strength $\lambda$ was chosen manually for best visual reconstruction performance.

#### Vectorcardiogram

VCGs were obtained at 0.75 T and 1.5 T from the ECG-triggering signal of a cardiac CINE acquisition to investigate the change in the magneto-hydrodynamic effect and potential implications for cardiac trigger accuracy [34]. ECG electrodes were placed on one healthy volunteer and the VCG measurements were performed in direct succession at the 0.75 T and 1.5 T MRIs. As cardiac CINE scans were acquired in parallel, the heart was located in the isocenter position.

### Dixon water/fat separation

Abdominal water/fat-separated imaging using multi-acquisition Cartesian and spiral Dixon approaches as well as a multi-echo Cartesian Dixon scan were performed in a coronal, abdominal slice [23, 31–37].

For the reconstruction of the spiral data, water/fat separation was performed in k-space. A seven-peak fat spectrum was employed to deblur the fat channel [38, 39]. Echo and water/fat images were reconstructed and then fed into an iterative reconstruction pipeline to estimate a B0 map from the phase of forward simulated and acquired echo images. Equivalent to the B0 map scans, the B0 map was first unwrapped and then blurred with the same adaptive Gaussian filter settings. Deblurring of all images was performed using multi-frequency interpolation [29] and the deblurred echo and water/fat images were then fed back into the B0 estimation code. For homogeneity correction, the first echo was blurred and used as a fake QBC reference image. Cartesian images were reconstructed equivalently, omitting spiral reconstruction and deblurring steps. Fat spectrum deblurring was performed using the 7-peak model directly in image space.

### Water/fat-separated MR fingerprinting

Water–fat-separated MR Fingerprinting based on the work of Koolstra et al. [40] was performed by adjusting echo times to the in- and out-of-phase echo times of water/fat at 0.75 T [24]. Each flip angle in a 500 time-point constant-TR FISP-MRF [41] sequence was acquired twice with 4.6 ms and 9.21 ms echo time in an interleaved fashion, resulting in a total of 1000 timepoints at a TR of 25 ms. A spiral acquisition with seven-fold undersampling was used. K-space data were separated into water and fat, demodulated for multi-frequency interpolation, and the fat channel was fat-spectrum deblurred using a seven-peak fat model [38, 39]. After transformation to image space, demodulation frequencies were recombined using multi-frequency interpolation [29] and a separately acquired B0 map (see above’s spiral Dixon protocol for details). A dictionary resolving *T*1 ([2:2:100, 100:10:1000, 1000:20:2000, 2000:40:5000] ms) and *T*2 ([2:2:150, 150:10:500, 500:20:1000, 1000:40:2000] ms; *T*2 < *T*1) for B1 + values between 50 and 80% in 5% steps was obtained using the extended phase graph [42, 43]. The dictionary was compressed using a sequentially truncated higher-order SVD with singular value thresholding (relative threshold 10^–4^) [44]. B1 + was prescribed during the matching step [45] by acquiring a co-registered B1 + map using DREAM (see Table 1, B1 + Mapping DREAM (for MRF)) [30]. The dictionaries were linearly interpolated to intermediate B1 + values by interpolation of the B1 + basis functions and subsequent multiplication with the core tensor and followed by dictionary normalization.

### Abdominal balanced SSFP and dual-TE GRE

A transverse volume in the abdomen of 15 slices was acquired using Cartesian balanced SSFP in multi-2D acquisition mode.

To assess water/fat separation using a dual-TE Dixon scheme [35], RF- and gradient-spoiled GRE was performed. The scan duration of 36 s was split over two breath-holds. Equivalent to the three-point Dixon method, water and fat images as well as a B0 map were obtained iteratively based on estimation of a B0 map from the difference of forward simulation and the acquired dual-TE images. In each iteration, the estimated B0 map was first unwrapped in 3D and then blurred in 3D using the same adaptive in-plane filter strength as in the 2D Dixon and B0 scans and a constant filter of 2 px in through-slice direction. The adaptive blurring was chosen to first obtain good separation of water and fat by promoting a smooth B0 map before fine-tuning the map to account for local field inhomogeneity. The updated B0 map was then used to refine the water/fat separation and the resulting forward simulation. Both forward simulation and the water/fat inference used a seven-peak fat-spectrum model [38, 39].

### Calf muscle spectroscopy

Point-resolved spectroscopy (PRESS) was performed in the calf muscle to compare proton metabolite spectra at 0.75 T, 1.5 T, and 3 T [20]. Spectra were acquired with 1 kHz, 2 kHz, and 4 kHz bandwidth at 0.75 T, 1.5 T, and 3 T, respectively, with 512 samples each, a TR of 2 s, in an 8 mL (10 × 20x40 mm^3^) voxel placed in the soleus muscle (Fig. 10a). Sixteen water-unsuppressed and 192 water-suppressed spectra, obtained with chemical-shift-based selective water suppression [46], were acquired. Power optimization was performed by observing the intensity of the water peak in a series of experiments with proportionally scaled excitation and echo pulses. Shimming was performed using a pencil beam and first-order shim settings. Spectral data were noise decorrelated, coil channel weights were obtained from water-unsuppressed averages and coil combination was performed using singular value decomposition [47]. Phase correction of the spectra was performed on the water peak for unsuppressed water spectra and on the main triglyceride peak at 1.3 ppm for water-suppressed spectra. Water-unsuppressed spectra were used to obtain frequency correction information, which was transferred onto water-suppressed spectra by averaging the water frequency shift. To perform eddy current correction, the average phase of the water-unsuppressed signal was subtracted from all signals.

Equal parameters within a group of scans were merged into a single row. *AQ* Acquisition, *2D* single slice, *M-2D* Multiple 2D scans, *MS* Interleaved Multi-Slice, *ME* Multi-Echo, *MA* Multi-Acquisition, *Rect.* *FOV* Rectangular Field-of-View, percentage in phase-encode direction, $\Delta TE$ Echo-time increment for multi-echo/multi-acquisition scans, *TE1/TE2* first/second echo time, No. BH × Dur. Number of breath-holds times duration per breath-hold, *Δ* Slice Gap

## Results

### System characterization

**Fig. 2:**
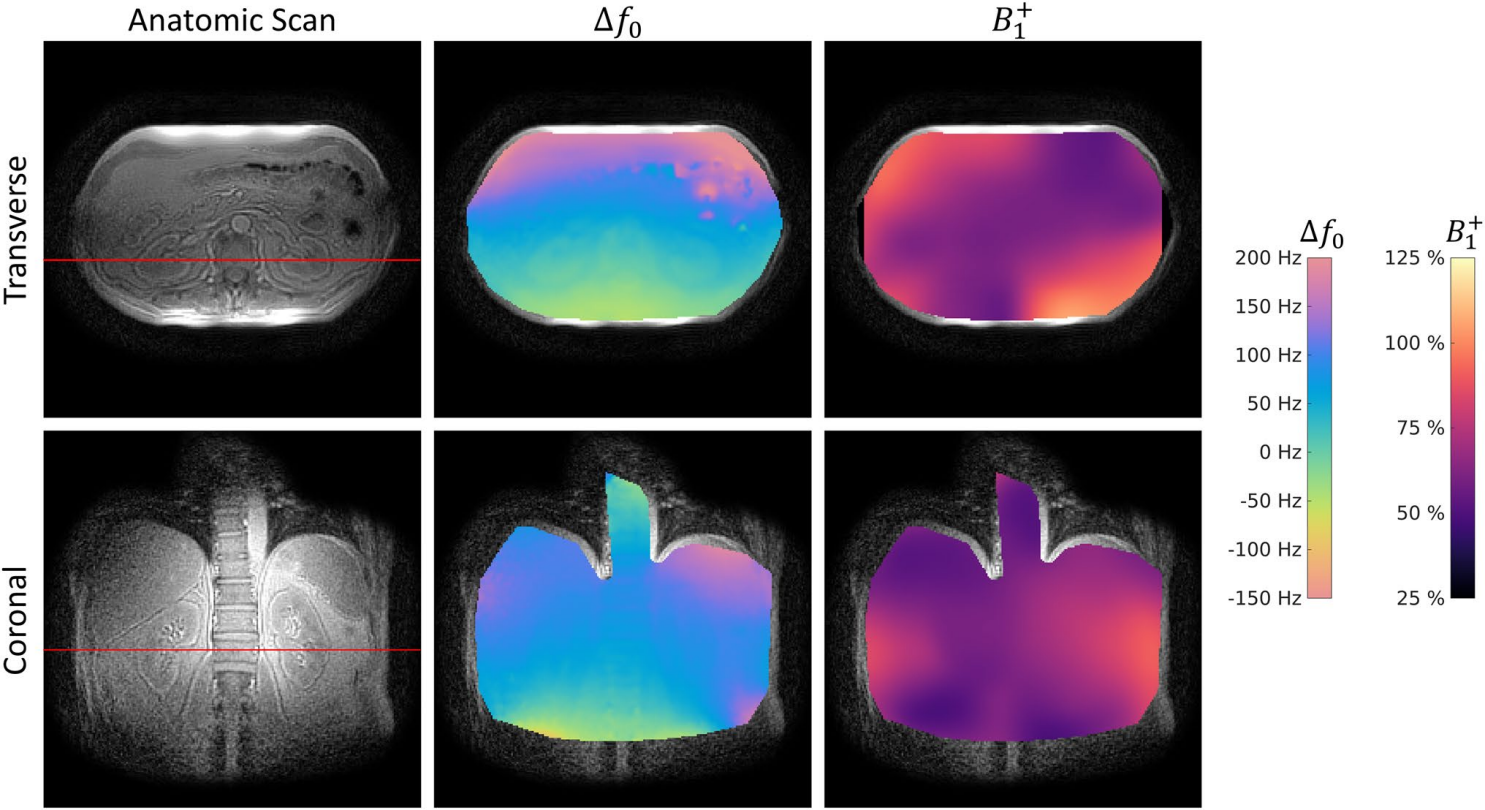
In vivo static-field and transmit-field inhomogeneity overlayed on an anatomical reference for transverse and coronal angulations. The red lines indicate the anatomical cross-reference of the two slices. NB: For transmitting, a custom-built dual-loop Helmholtz transmitter (previously used for ^13^C experiments at 3 T) is used at 0.75 T, not a body coil. In the transverse orientation, a linear gradient of approximately 150 Hz in anterior–posterior direction is seen. B1 + shows a pronounced drop in transmit efficiency to 50% in the center of the body and only reaches prescribed flip angles in left- and right-posterior regions. In the coronal angulation, both B0 and B1 + appear more homogenous. However, a maximum transmit efficiency of 80% is reached in the spleen and kidney

Figure  2 shows in vivo B0 and B1 + mapping results alongside an anatomical reference. In a transverse slice, a linear field gradient of approximately 150 Hz between anterior and posterior positions is observed. B1 + was found to be inhomogeneous with only 50% transmit efficiency in the center of the transverse slice and higher than nominal tip-angles in the left- and right-posterior regions. In the coronal slice, the B0 map appears more homogenous, however, also shows up to 100 Hz frequency difference between the spine and the liver, spleen, and kidneys. B1 + mapping results are equally more homogenous reaching approximately 80% transmit efficiency in the left kidney and spleen compared with approximately 60% in the liver and right kidney.

**Fig. 3:**
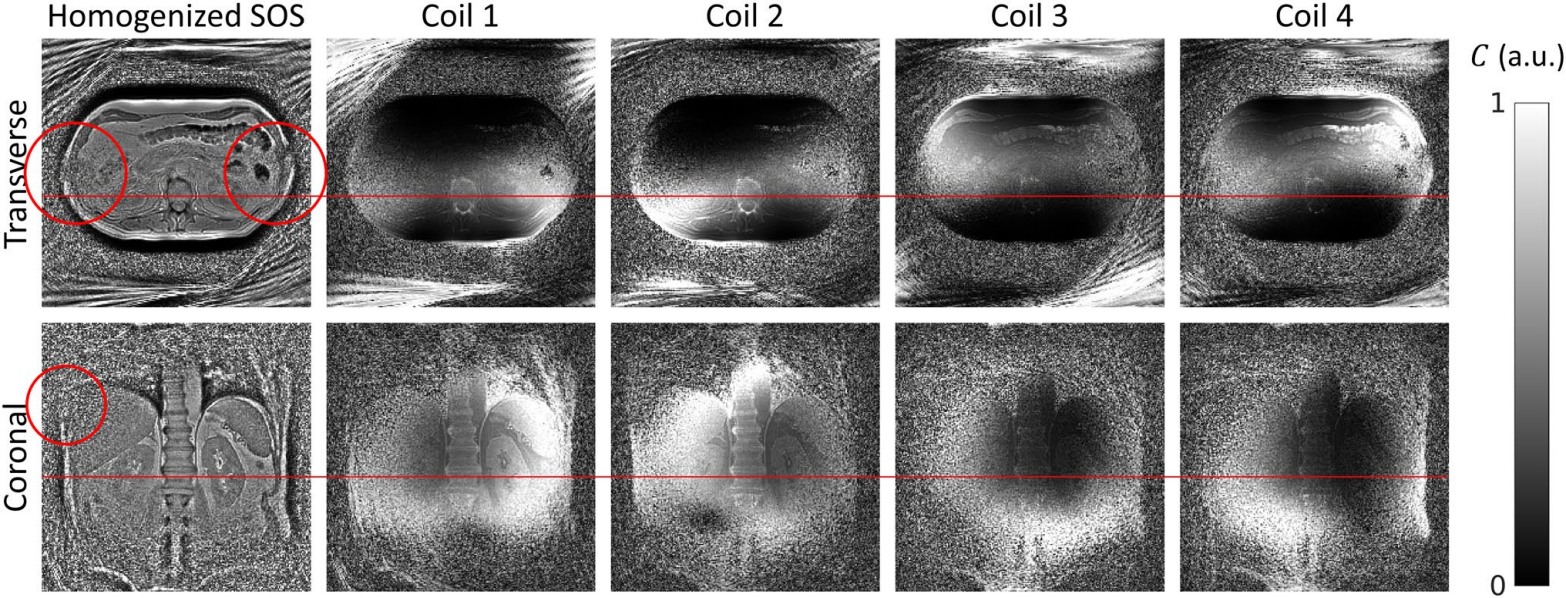
(Left Column) Homogenized sum-of-squares (SOS) reconstruction. (Column 2 to 5) Single coil element reconstructions divided by their SOS for the coronal and transverse orientations. The red lines indicate the anatomical cross-reference of the two slices. Due to the relatively small size of the receive elements (7.6 cm × 18.3 cm), the penetration depth is reduced leading to amplification of noise in the center of the body in the transverse orientation (red circles). In the coronal slice, the anterior elements (coil 3 and coil 4) contribute little signal

Figure  3 shows single-coil element reconstructions of the B0 scans for both the transverse and the coronal orientation. Due to the relatively small size of the receive coil elements (7.6 cm × 18.3 cm), their penetration depth and lateral field of view is limited, which leads to elevated noise in the homogenized sum-of-squares image (left column), especially in the lateral regions of the transverse slice (red circles).

**Fig. 4:**
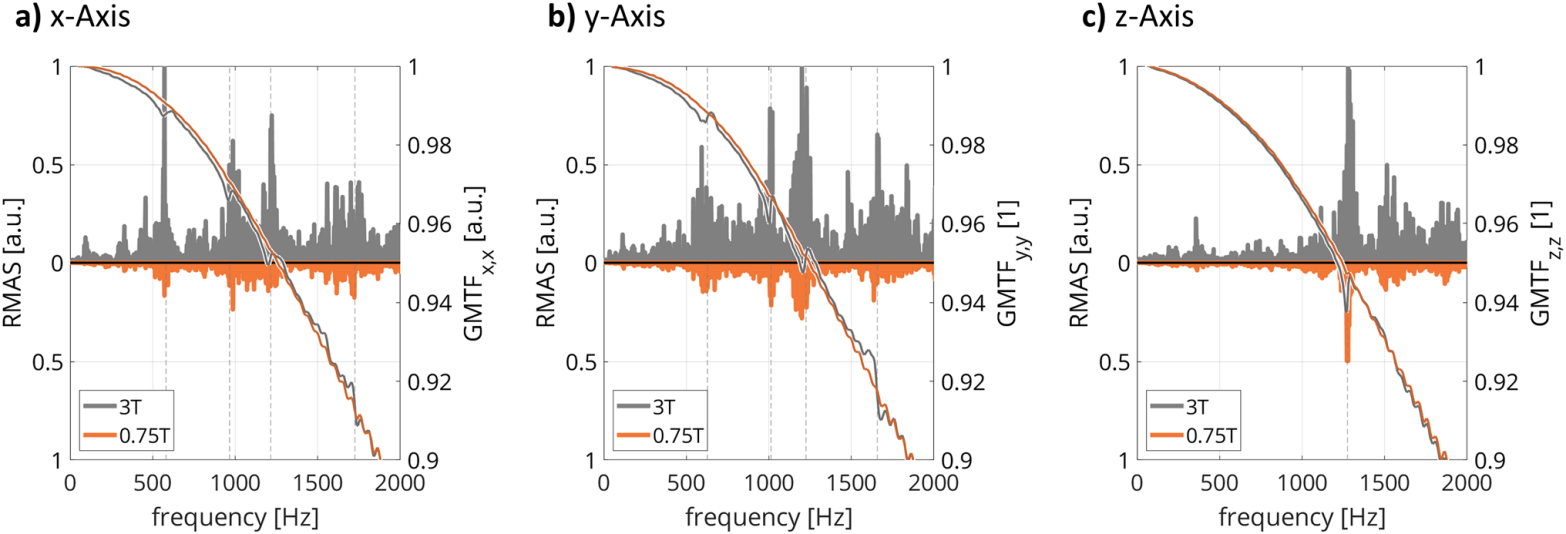
Overlay of the first-order gradient modulation transfer function (GMTF; line plots) on relative microphone amplitude spectra (RMAS; filled curves) of the system in product configuration (gray) and the lower-field configuration (orange) for the three gradient axes (**a**, **b**, **c**). Mechanical resonances are marked by dashed vertical lines. A pronounced reduction in mechanical resonances is observed in the GMTF (e.g., at approximately 1300 Hz on the z-axis), which is accompanied by a reduction in sound pressure. Figure adapted from Ref. [17]

In Fig. 4, the gradient modulation transfer function (GMTF) of the *x*-, *y*-, and *z*-axis gradients for the same system operated at 0.75 T (orange) and 3 T (gray) is depicted together with relative microphone amplitude spectra (RMAS) obtained from concurrent audio measurements. At lower field, mechanical resonances are reduced well below the uncertainty threshold of the thin-slice acquisition technique, while acoustic noise is significantly reduced over the whole frequency spectrum.

### In vivo showcases

**Fig. 5:**
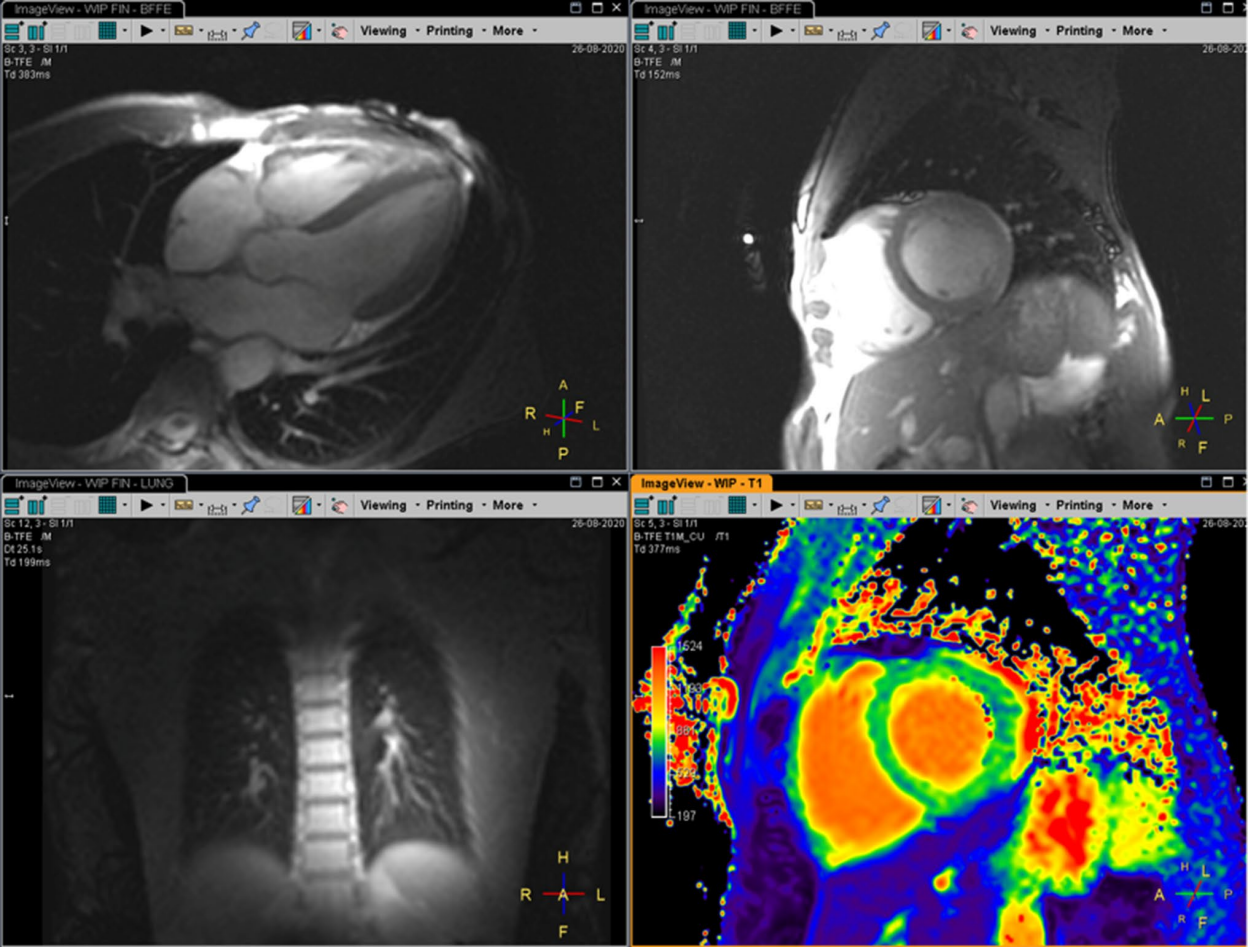
Screenshot of the vendor’s user interface showing reconstructions performed on the system. Top left: cardiac cine four-chamber view. Top right: cardiac cine short-axis view. Bottom left: free-breathing balanced SSFP of the lung. Bottom right: cardiac T1 mapping

Figure  5 shows a screenshot of the vendor’s user interface of a cardiac cine scan in short-axis and four-chamber orientations, a lung bSSFP, and a cardiac T1 mapping scan. All images were reconstructed directly using the vendor’s reconstructor. Due to the missing body coil data, contrast equilibration was not possible leading to pronounced hyperintensities, e.g., in the right ventricle of the short-axis view, when compared with the left ventricle. Apart from contrast inhomogeneity, the reconstruction quality is good and allows judging overall scan quality as well as general scan planning.

**Fig. 6:**
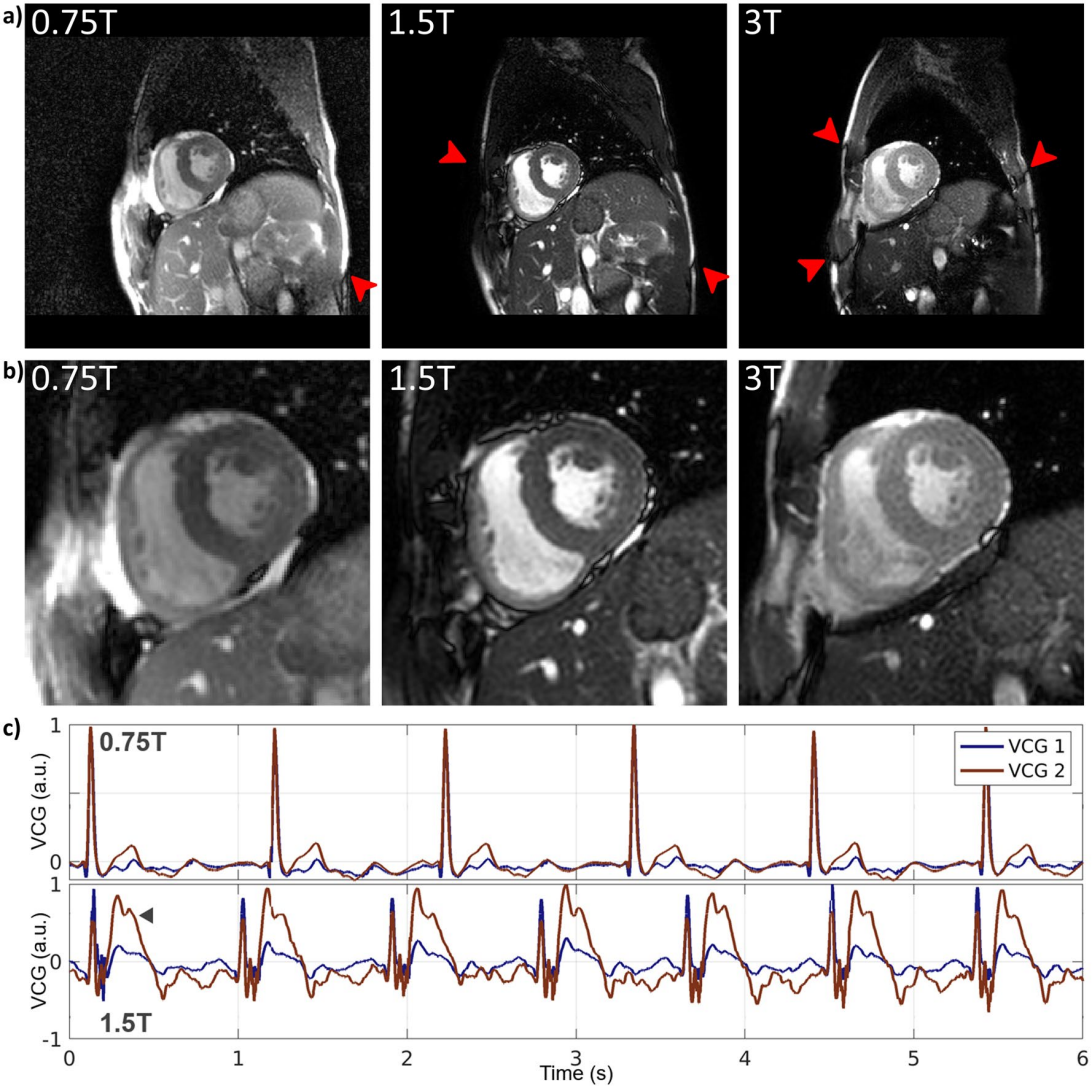
**a** Comparison of short-axis cardiac cine images of the same volunteer obtained at 0.75 T, 1.5 T, and 3 T with **b** zoom-ins of the heart. Red arrows indicate banding artifacts. Compared to 1.5 T and 3 T, the 0.75 T image is nearly banding artifact free due to the improved field homogeneity relative to the scans repetition time. **c** Vector cardiogram (VCG) traces of the same volunteer with equal electrode placement obtained at the 0.75 T system (top row) and the 1.5 T (bottom row). The arrow marks the magneto-hydrodynamic effect, which is of equal magnitude as the R-wave on a 1.5 T system for derivation 2 (red curve) and is nearly completely missing on the 0.75 T system. Hence, higher trigger accuracy is expected for cardiac scans on a lower-field system

In Fig. 6a, a comparison of cardiac short-axis cine scans in peak systole for the same volunteer at 0.75 T, 1.5 T, and 3 T is shown, with Fig. 6b showing zoom-ins of the heart. The myocardium, lumen and papillary muscles can be well delineated in all scans. Contrary to 1.5 T and 3 T, the 0.75 T cine image is devoid of banding artifacts as field homogeneity in relation to the employed repetition time is improved. In Supporting Video 1, a comparison of retrospectively undersampled cardiac cine short-axis, four-chamber, and long-axis views are shown for acceleration factors of 2, 4, and 8 corresponding to breath-hold durations of 8, 4, and 2 s, respectively.

Figure 6c shows a comparison of vector cardiogram (VCG) traces at 0.75 and 1.5 T for the same volunteer with unchanged electrode placement. Due to the reduced field, the magneto-hydrodynamic effect is greatly reduced at 0.75 T, while it can easily surpass the voltage of the R-wave on a 1.5 T system.

**Fig. 7:**
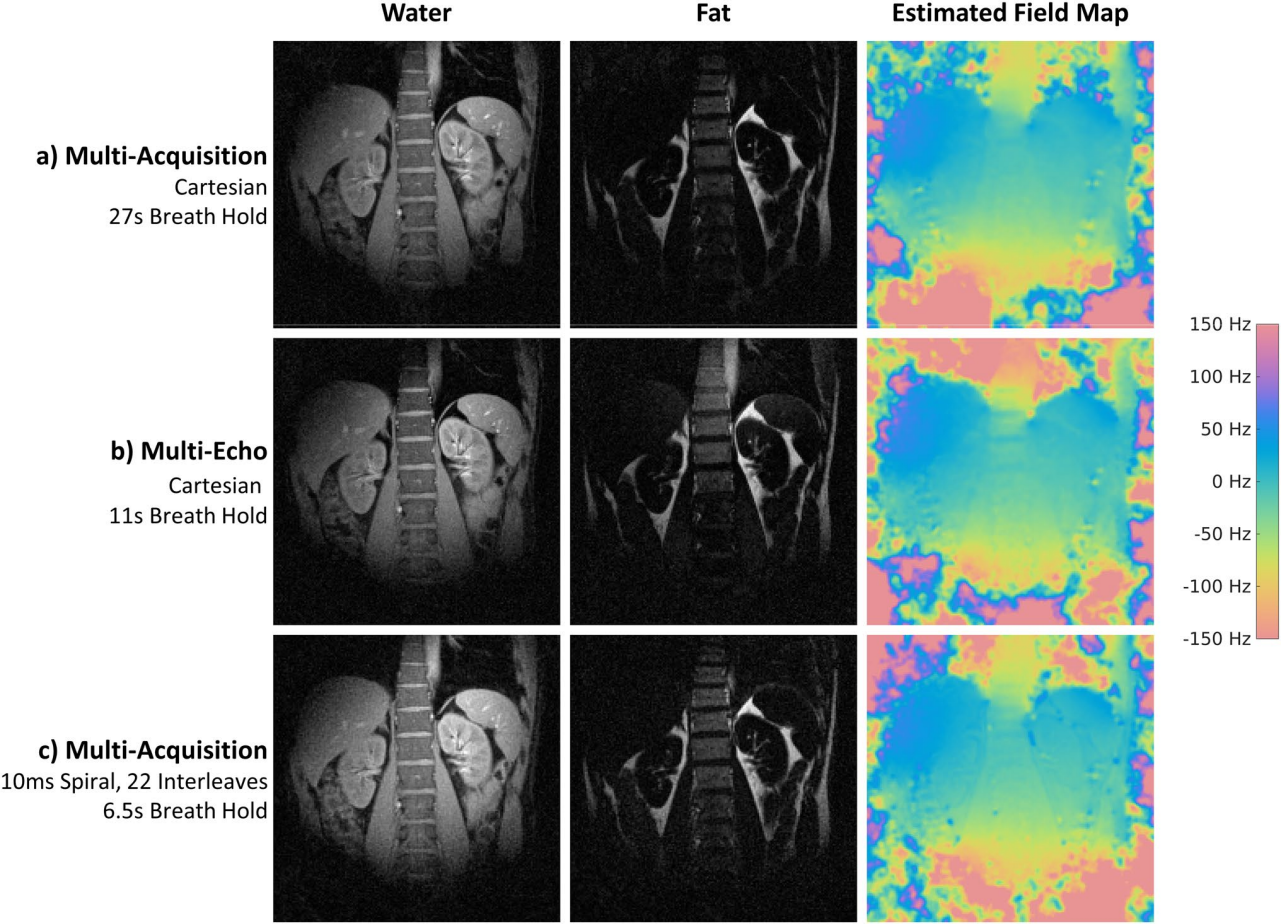
Comparison of coronal water/fat separation using the three-point Dixon method. **a** A multi-acquisition Cartesian, **b** a multi-echo Cartesian, and **c** multi-acquisition spiral mode is shown. Depicted are the reconstructed water and fat maps as well as an iteratively determined B0 map

Figure 7 compares water/fat separation results obtained with a three-point Dixon method with multi-acquisition Cartesian, multi-echo Cartesian, and multi-acquisition spiral readouts. The water/fat images as well as the iteratively determined B0 maps are in good agreement. Using spiral imaging, the breath-hold duration could be reduced from 27 s to 6.5 s for a single slice.

**Fig. 8:**
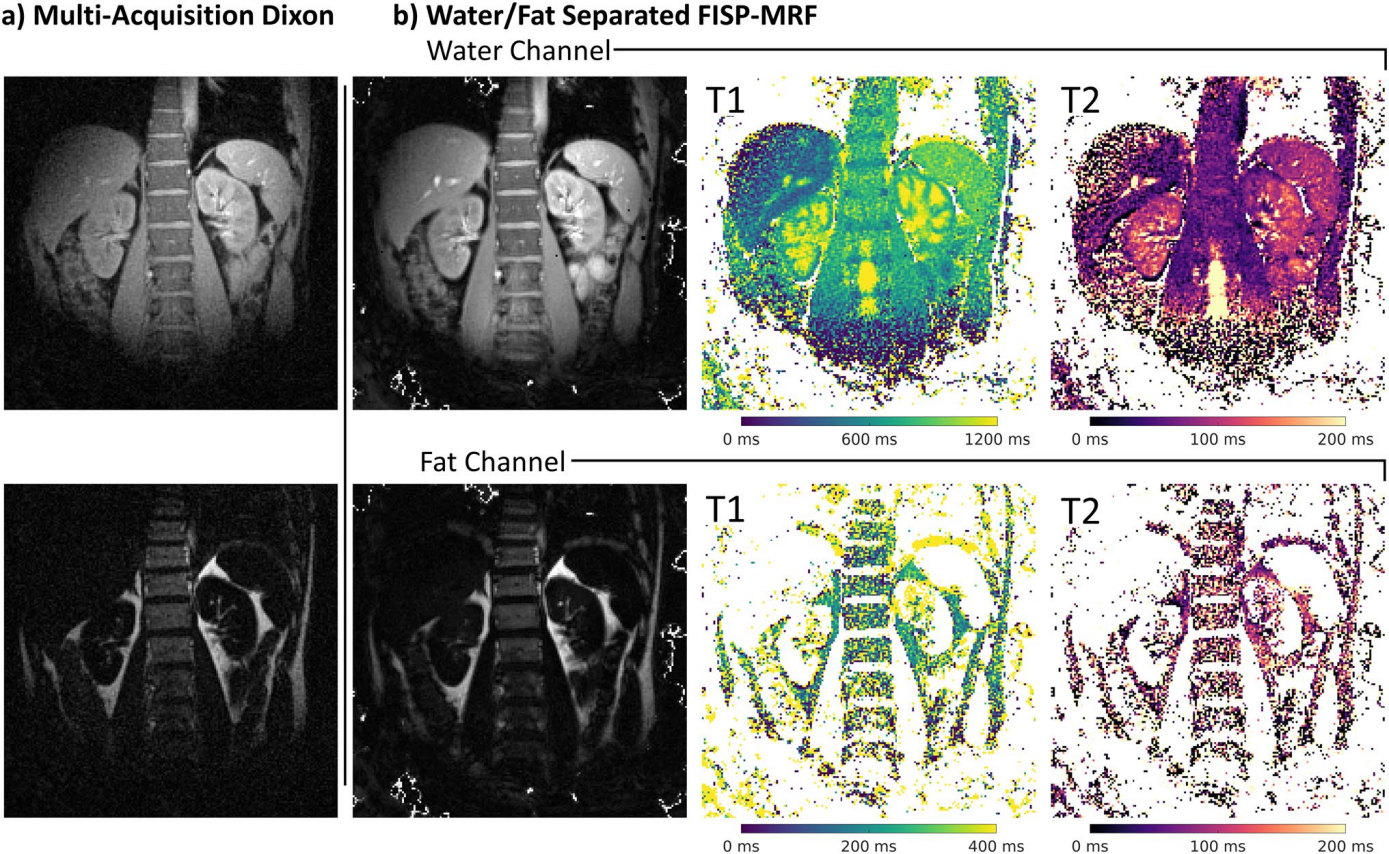
Water/fat-separated quantitative parameter maps obtained using MR Fingerprinting. **a** Multi-acquisition spiral Dixon scan for reference. **b** Water/fat-separated FISP-MRF images showing the reconstructed water and fat map in the first column followed by *T*1 and *T*2 matching results. Approximate relaxation parameters are (mean ± standard deviation over region of interest; data from one volunteer only): liver (*T*1: 491 ms ± 188 ms; *T*2: 77 ms ± 97 ms), spleen (*T*1: 911 ms ± 84 ms; *T*2: 91 ms ± 24 ms), kidney (*T*1: 958 ms ± 206 ms; *T*2: 111 ms ± 50 ms), muscle (*T*1: 744 ms ± 94 ms; *T*2: 50 ms ± 13 ms), and fat (*T*1: 195 ms ± 104 ms; *T*2: 105 ms ± 81 ms)

In Fig. 8, proton density, T1 and T2 maps from water/fat-separated MR Fingerprinting are shown and compared to the water/fat separation from the previous multi-acquisition spiral approach. The water/fat-separated proton density maps are in general agreement with the multi-acquisition Dixon scan and show less pronounced noise.

**Fig. 9:**
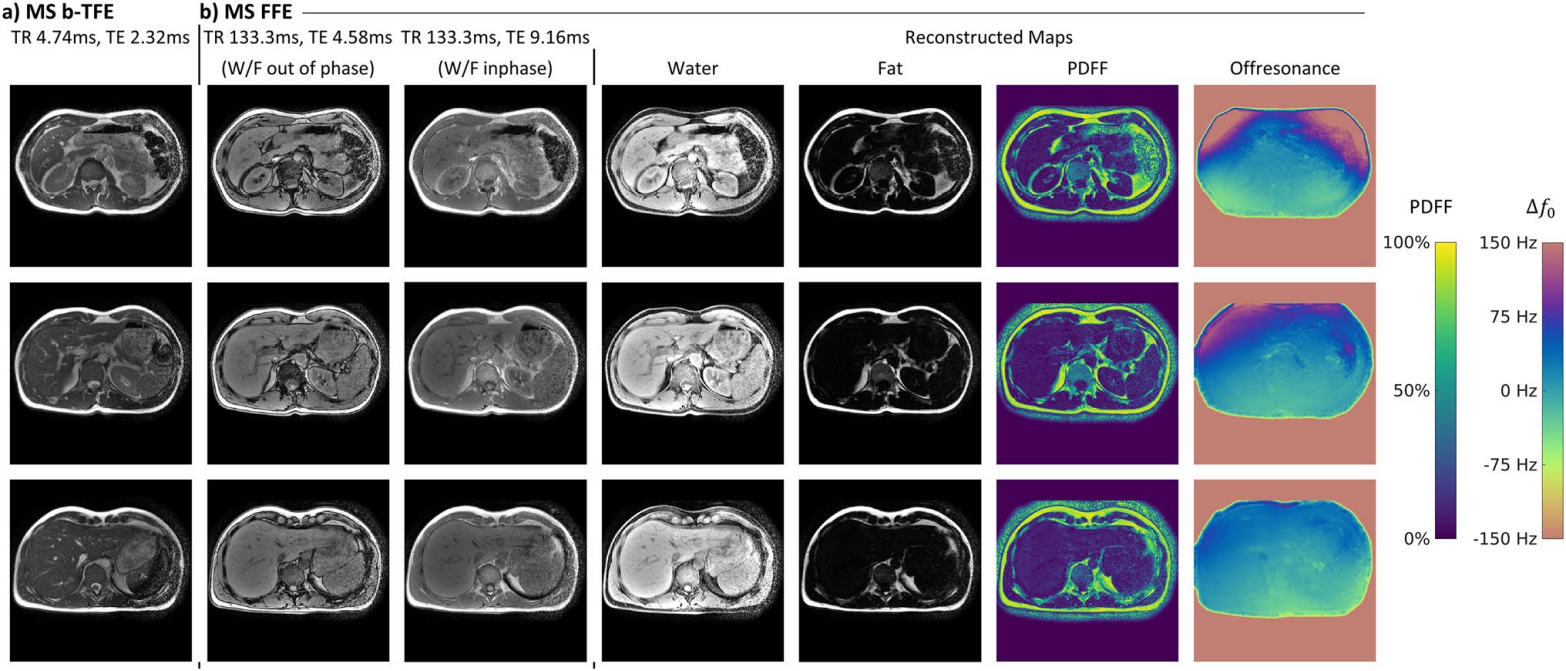
**a** Single breath-hold, volumetric balanced SSFP of the abdomen, showing three exemplary slices. **b** Multi-slice dual-TE spoiled GRE with water/fat in- and out-of-phase echo times and iteratively reconstructed water/fat, proton density fat fraction, and off-resonance maps

In Fig. 9a, three exemplary slices of a single breath-hold, balanced SSFP acquisition in the abdomen are given, showing good soft-tissue and vessel contrast as well as no banding artifacts. Figure 9b shows the same slices acquired with a RF- and gradient-spoiled GRE sequence with water/fat in- and out-of-phase echo times. Using the complex dual-TE images, an iterative water/fat separation with B0 estimation could be performed with results shown on the right of Fig. 9b. Water and fat could be successfully separated despite the strong field gradient in anterior–posterior direction leading to approximately 150 Hz frequency shift. In comparison, the water/fat frequency shift at 0.75 T is approximately 108 Hz.

**Fig. 10:**
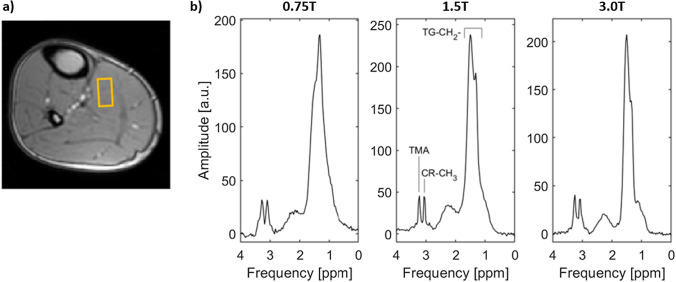
**a** Localizer image of the calf muscle with overlayed location of the 8 mL PRESS voxel in the soleus muscle. **b** Comparison of 0.75 T, 1.5 T, and 3 T water-suppressed PRESS spectra showing triglyceride (TG-CH_2_), creatine (CR-CH_3_), and trimethylammonium (TMA). Figure adapted from Ref. [20]

In Fig. 10, water-suppressed PRESS spectra of the same volunteer are shown for 0.75 T, 1.5 T, and 3 T next to a localizer image with the location of the voxel in the calf muscle. Despite the lower field strength, the main metabolite peaks of triglyceride (TG-CH_2_), creatine (CR-CH_3_), and trimethylammonium (TMA) can be resolved even though absolute frequency shifts between the metabolites are reduced. This is due to the proportional reduction in line width due to longer T_2_* times at lower field.

## Discussion

In this work, we have demonstrated that a clinical 3 T system can be temporarily ramped down to 0.75 T and be used in conjunction with existing ^13^C transmit and receive hardware to study and compare MRI and MRS on the very same system with limited time and cost overhead. Clinical and research protocols could be readily executed at 0.75 T including image and spectroscopy data reconstruction. Additional offline reconstruction allowed us to study data in detail and to improve image and spectral quality, as coil sensitivities could be estimated. This enabled to take phase differences between the receive elements into account as well as gave the possibility to perform contrast equalization [48] by emulating a low-resolution body coil image and using it for QBC-regularized reconstruction reducing surface coil flare and leading to similar contrasts and image homogeneity as on clinical systems.

Due to the 3 T factory shims bolted onto the cryostat and the limited available space on the insertable shim rails to compensate them, static-field homogeneity was lower than expected and did not reach industry standards of ≤ 3 ppm for the 50 × 50 × 45 cm^3^ volume. Together with the limited automatic shimming accuracy in vivo, which was due to the lack of a body coil with homogenous transmit and receive fields, this led to reduced overall shim quality with, e.g., a frequency difference of approximately 200 Hz in anterior–posterior direction in abdominal scans. Additionally, the dual-loop Helmholtz transmitter created an inhomogeneous transmit field which, in combination with the low-power multi-nuclear amplifier (4 kW), limited achievable flip angles in vivo and further reduced contrast homogeneity. Quantitative protocols, such as MRF, were feasible, but required B1 + correction due to the custom-built transmit coil. In addition, the relatively small size of the four-channel receive coils led to a small combined field of view, which in a transverse orientation in the abdomen led to pronounced noise enhancement in lateral areas half-way between anterior and posterior of the subjects.

Regarding the limited shim quality, it is noted that main field shimming on more modern clinical scanners is exclusively performed using insertable shim rails (avoiding bolted shim irons altogether), thereby providing more flexibility to shim the same magnet at different field strengths. Also, full- or reduced-rung ^13^C body resonators are becoming available to allow addressing the limitations of limited transmit-field homogeneity. In this regard, ramp-down experiments are also considered valuable in assessing ^13^C transmit and receive coil performance, which otherwise can only be performed with limitations on enriched and expensive ^13^C samples. Using the same rationale, transmit/receive hardware for imaging other nuclei can be repurposed for proton imaging as well. Table 2 shows a collection of typical MR nuclei and how they translate to equivalent proton field strengths. E.g., ^129^Xe coils could be used for imaging protons at 0.83 T. ^13^C corresponds to the lowest field strength officially supported by the vendor’s multi-nuclear MR product.

**Table 2:** List of nuclei, their reduced gyromagnetic ratio, and their equivalent field strength, if proton imaging was performed by ramping down a 3 T MRI system to match Larmor frequencies

Nuclei	Reduced gyromagnetic ratio $\rlap{--} \gamma$(MHz/T)	Ratio ${{\rlap{--} \gamma _{{\text{x}}} } \mathord{\left/ {\vphantom {{\rlap{--} \gamma _{{\text{x}}} } {\rlap{--} \gamma _{{1{\text{H}}}} }}} \right. \kern-\nulldelimiterspace} {\rlap{--} \gamma _{{1{\text{H}}}} }}$	Equivalent field strength for proton imaging
^1^H*	42.577	100%	3.00 T
^19^F	40.078	94.13%	2.82 T
^3^He	− 32.432	76.17%	2.29 T
^31^P*	17.235	40.48%	1.21 T
^7^Li	16.545	38.86%	1.17 T
^129^Xe*	− 11.777	27.66%	0.83 T
^23^Na*	11.262	26.45%	0.79 T
^13^C*	10.708	25.15%	0.75 T
^17^O	− 5.771	13.55%	0.41 T

*Listed as supported by the vendor for multi-nuclear MR at 3 T

Acquisition of the gradient modulation transfer function for both the product and the lower field strength allowed to conclude that lower field features improved gradient performance, especially since mechanical resonances are reduced due to a proportional reduction in Lorentz forces. This was also confirmed by a reduction in sound pressure, which benefits image quality and patient comfort.

The magneto-hydrodynamic (MHD) effect in the VCG signal was found to be reduced at 0.75 T compared with 1.5 T. This directly leads to more robust cardiac triggering and hence directly benefits cardiac MR applications.

Feasibility of water/fat separation with both multi-acquisition and multi-echo approaches using spiral and Cartesian acquisitions was demonstrated. In addition, we performed water/fat-separated MR fingerprinting, which showed comparable performance in separating water and fat and in addition delivered separate *T*1/*T*2 maps. Comparing liver *T*1/*T*2 values to 0.55 T, our values (mean ± std. dev. over region of interest *T*1: 491 ms ± 188 ms; *T*2: 77 ms ± 97 ms) show longer T1 and *T*2 values as compared to 0.55 T (*T*1: 359.9 ms, *T*2: 45.2 ms) [49]. While a prolongation of *T*1 with increasing field strength is expected, the increase in T2 is unexpected. However, given the relatively high standard deviation over the ROI in both *T*1 and *T*2 estimates, our quantitative findings should be interpreted with caution. Comparing our spiral MRF results to similar work at 0.55 T [49], the benefit of fat-spectrum deblurring should be noted, which lead in our work to well-delineated water/fat boundaries and little blurring of the visceral fat despite a comparable spiral acquisition duration.

The in vivo calf muscle experiments show that spectroscopy is feasible and utilizable at lower fields and despite the proportional lowering of line separation, peak separation can be maintained as line width is decreasing due to favorable scaling of T2*. Similar to the imaging experiments, an improvement of shim and transmit-field homogeneity would be beneficial for further reducing line width and hence further improving peak separation and, thus, utility of spectroscopy at 0.75 T [20].

We claimed the system to be low cost compared to the purchase and installation of a new lower-field MRI system. Comparing our costs of approximately 100 k CHF (including investments and initial pulse-programming/development costs) to the purchase of a new lower-field system—likely being in the 1 M CHF range without siting related costs—ramp-down of an existing system, especially if transmit/receive hardware is already available, is a viable, cost-effective option.

The overall experience of ramping down a high-field MRI was experimental as we faced many issues that have been solved on clinical systems with methods in place that completely shield the high-level user from their existence, e.g., automatic receive gain adjustment to prevent ADC overflow. These issues were reexperienced as measures normally active on a clinical system were not operational due to the lack of tuned body and pickup coils. Despite these challenges, it was a great learning experience especially with regards to the fundamental mechanisms of MRI and the tremendous engineering effort that has been put into state-of-the-art machines. A body coil tuned to 0.75 T would have been of enormous help for both transmit homogeneity and making preparation phases work. Purchasing a volume transmitter coil to improve transmit homogeneity was also considered, however, as our system has a narrow bore (60 cm), only limited space remains for additional transmit hardware. In hindsight, it would have also been helpful to carefully study other experimental systems that also operate without a body coil, such as the Philips 7 T Achieva, to transfer preparation phases and pulse-programming features. Additionally, there are many user-hidden variables that optimize the scanner for a particular field strength. It is important to spend exclusive time to ensure these are set properly for low-field operation.

## Conclusion

Clinical 3 T systems can be temporarily ramped down and operated at 0.75 T by exploiting ^13^C transmit/receive hardware allowing to run and reconstruct clinical and research protocols with minimal software modifications. This approach allows to study and compare MRI and MRS application on the very same system with limited time and cost overhead.

## Supplementary Information

Below is the link to the electronic supplementary material.

Supplementary file1 (DOCX 1019 KB)

Supplementary file2 (AVI 12556 KB)

## Data Availability

The full MATLAB code, raw datasets as well as pre-processed MRI data are openly available for download from: https://gitlab.ethz.ch/ibt-cmr-public/recon-0.75t-mri.
