# Supplementary material for: Ramping down a clinical 3 T scanner: a journey into MRI and MRS at 0.75 T
Source: MAGMA. 2023 May 12;36(3):355–73. doi: 10.1007/s10334-023-01089-9 (PMC10386956; doi:10.1007/s10334-023-01089-9)
Supplement: Supplementary file 1 — Supplementary file1 (DOCX 1019 KB) [file 10334_2023_1089_MOESM1_ESM.docx]

# Supporting Material


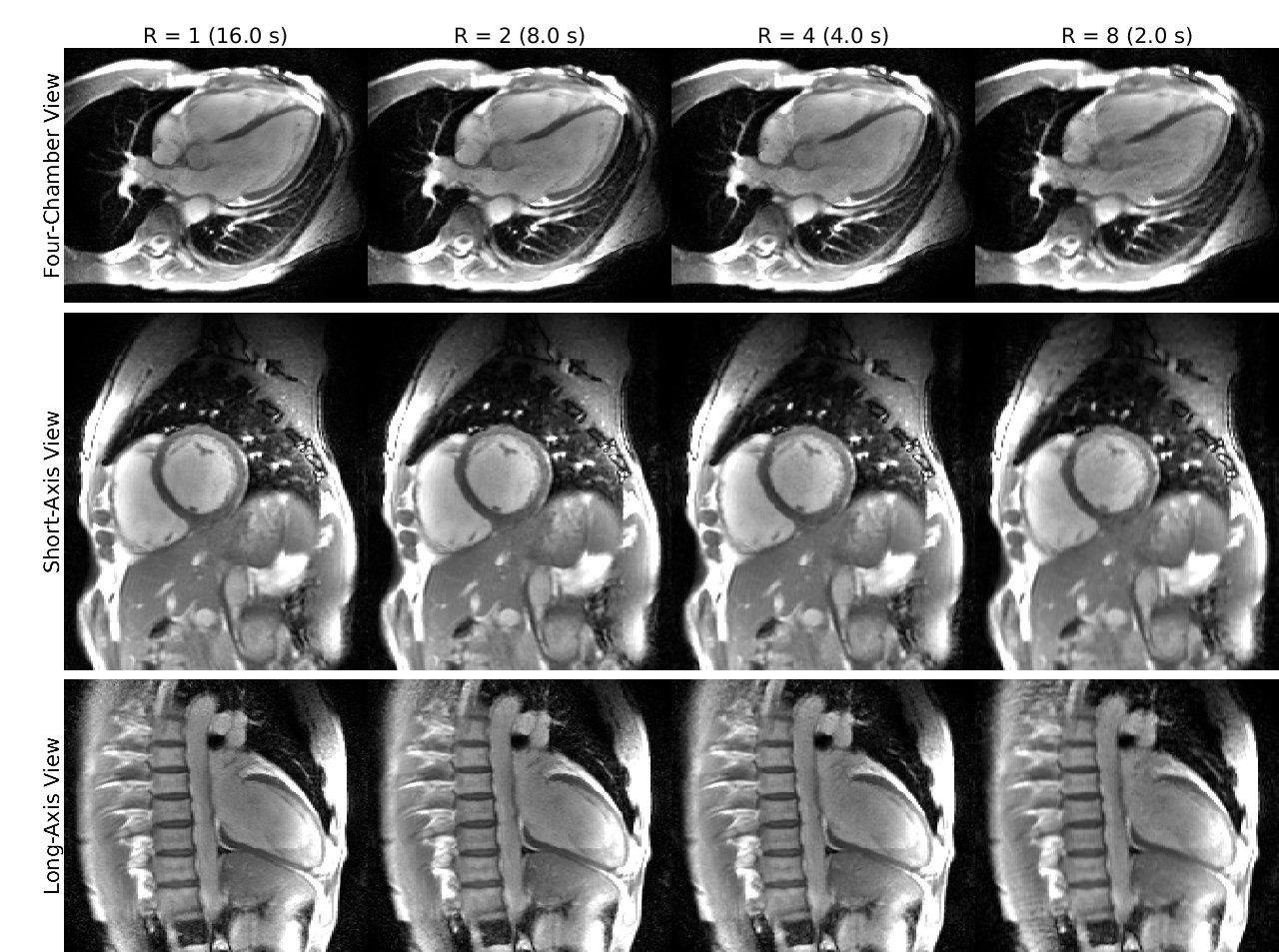


Supplementary Video 1: Cardiac cine scan in four-chamber, short-axis, and long-axis angulations accelerated by 2-, 4-, and 8-fold variable density undersampling and reconstructed using a vectorial total variation-regularized compressed sensing reconstruction.
